# The relationship between red blood cell distribution width at admission and post-stroke fatigue in the acute phase of acute ischemic stroke

**DOI:** 10.3389/fneur.2022.922823

**Published:** 2022-07-29

**Authors:** Meidi Peng, Yupei Chen, Yan Chen, Koulan Feng, Haiyan Shen, Hongtao Huang, Wenxuan Zhao, Hua Zou, Jianan Ji

**Affiliations:** ^1^School of Medicine (School of Nursing), Nantong University, Nantong, China; ^2^Department of Neurology, Nantong Third People's Hospital, Nantong University, Nantong, China; ^3^Department of Gerontology, Nantong Third People's Hospital, Nantong University, Nantong, China

**Keywords:** acute ischemic stroke, post-stroke fatigue, red blood cell distribution width, restricted cubic spline, biomarker

## Abstract

**Introduction:**

Post-stroke fatigue (PSF) is a common complication in the patients with acute ischemic stroke (AIS). This prospective study aimed to investigate the relationship between red blood cell distribution width (RDW) at admission and PSF in the acute phase.

**Methods:**

The AIS patients were enrolled in Nantong Third People's Hospital, consecutively. PSF in the acute phase was scored according to the Fatigue Severity Scale. Levels of RDW were measured at admission. The associations were analyzed using multivariate regression and restricted cubic splines (RCS).

**Results:**

From April 2021 to March 2022, a total of 206 AIS patients (mean age, 69.3 ± 10.7 years; 52.9% men) were recruited. After the adjustment for potential confounding factors, RDW at admission remained the independent associated factor with PSF in the acute phase (OR [odds ratio], 1.635; 95% CI [confidence interval], 1.153–2.318; *P* = 0.006). The linear dose-response associations of RDW with PSF in the acute phase were found, based on the RCS model (*P* for non-linearity = 0.372; *P* for linearity = 0.037). These results remained significant in other models.

**Conclusions:**

RDW at admission could serve as a novel biomarker of PSF in the acute phase of AIS.

## Introduction

Stroke is one of the vital reasons for global disease burden (1–3). Acute ischemic stroke (AIS) accounts for the vast majority of stroke. Although there are many treatment options for ischemic stroke nowadays (4–6), the AIS patients may undergo several complications during their treatment and rehabilitation. Post-stroke fatigue (PSF), whose incidence range from 23 to 85%, is a common emotional complication after the onset of AIS (7–11). PSF could lead to poor recovery and prognosis (7–11). Therefore, it is meaningful and fundamental to explore the related factors to PSF.

Red blood cell distribution width (RDW), which could be obtained from routine blood testing, is an easily accessible biomarker. According to previous studies, RDW is able to reflect the variability in volumes of peripheral red blood cells and associated with inflammation (12, 13). In addition, RDW has been shown to be an effective biomarker for many diseases. For instance, RDW may serve as an undesirable prognostic factor in patients treated with hematopoietic stem cell transplantation (14). The levels of RDW might be associated with long-term all-cause mortality in the patients with acute myocardial infarction (15). Moreover, one recent study, which is carried out by Li Y et al., have found that elevated levels of RDW at admission may be able to predict depression after the onset of AIS (16).

Nevertheless, to our knowledge, there is no research about the relationship between the levels of RDW at admission and PSF in AIS patients. Hence, our prospective study was designed to assess the role of RDW on PSF in the acute phase.

## Materials and methods

### Study subjects

From April 2021 to March 2022, the AIS patients were enrolled in our study from the Nantong Third People's Hospital, consecutively and prospectively.

The inclusion criteria were listed as follows:

- Onset within 7 days;
- Age≥18 years;
- With sufficient cognitive ability;
- With sufficient knowledge of the Mandarin Chinese language and Nantong local dialect.

The exclusion criteria were listed as follows:

- Pre-stroke fatigue;
- Incomplete data;
- Refuse to participate in the research;
- Poor mental state.

This study was registered with China clinical trial registration center (Registration number: ChiCTR2100044165), approved by the ethics committee of Nantong Third People's hospital (Ethics number: EK2021008), and performed according to the principles of the Declaration of Helsinki.

### Clinical assessments

The clinical assessments were completed in a separate conversation room. We collected general demographic questionnaires (age and sex), past medical history (hypertension, diabetes, cancer, coronary heart disease, atrial fibrillation, arthritis, previous stroke, alcohol abuse and tobacco use), clinical data (systolic blood pressure, diastolic blood pressure, stroke severity and anxiety severity) and laboratory parameters (fast blood glucose, total cholesterol, triglycerides, high density lipoprotein, low density lipoprotein, creatinine and RDW). Stroke severity was assessed by National Institute of Health stroke scale (NIHSS). Anxiety severity was assessed *via* Hamilton Anxiety Scale (HAMA). The levels of RDW at admission were measured within 24 h after admission.

### The definition of PSF in the acute phase

PSF was assessed by the Fatigue Severity Scale (FSS) within 2 weeks after onset of ischemic stroke. FSS consists of 9 items, each item according to the patient's evaluation of fatigue severity will gradually transition the result to 1–7 points. The higher the score, the more severe PSF. We took the total score of 36 as the dividing line (7).

### Statistical analysis

R software (Version 4.1.3; http://www.r-project.org) was used to conduct statistical analyses. Categorical variables are expressed as n (percentages). Normally distributed variables are expressed as the mean ± SD, and abnormally distributed continuous variables are expressed as medians (interquartile range [IQR]). The differences between PSF group and non-PSF group were identified with the Student's *t-*test, the Wilcoxon W-test, the chi-square test or Fisher's exact test as appropriate. The violin plot was utilized to present the distribution of RDW between the PSF group and the non-PSF group. We explored the relationship between RDW and PSF in different logistic regression models. Model 1 was unadjusted model. Model 2 was adjusted for age and sex. Model 3 was adjusted for age, sex, coronary heart disease and baseline NIHSS score. Model 4 was adjusted for age, sex, coronary heart disease, NIHSS score and HAMA score. What is more, we used restricted cubic splines (RCS) with three knots placed at the 10th, 50th, and 90th percentiles to evaluate the dose-response relationship of RDW with PSF in different models. *P* < 0.05 was considered statistically significant.

## Results

**Figure 1:**
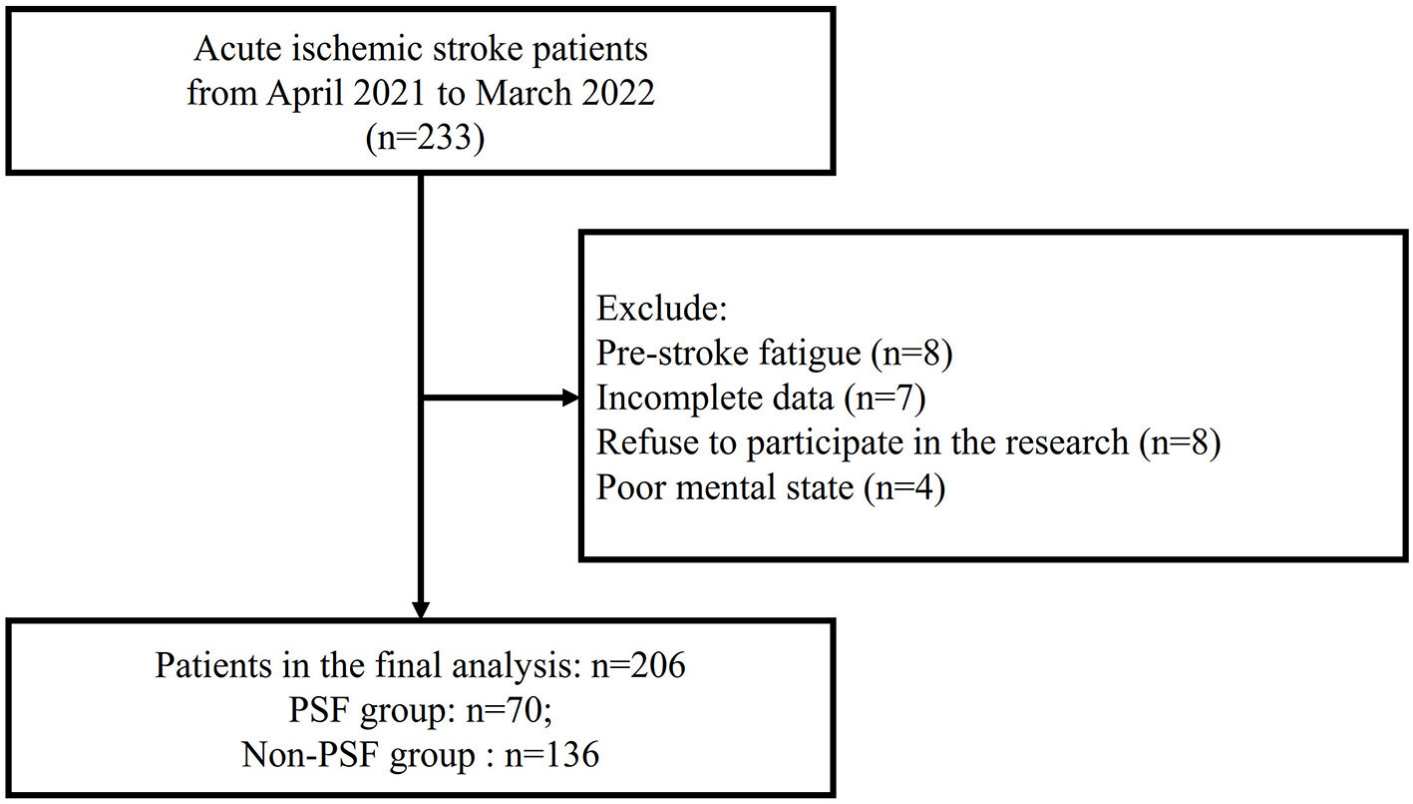
Flowchart.

From April 2021 to March 2022, we screened 233 AIS patients, and excluded 27 AIS patients as following reasons: Pre-stroke fatigue (*n* = 8); Incomplete data (*n* = 7); Refuse to participate in the research (*n* = 8); Poor mental state (*n* = 4) (Figure 1). Finally, a total of 206 AIS patients (mean age, 69.3 ± 10.7 years; 52.9% men) were included in the analysis.

**Figure 2:**
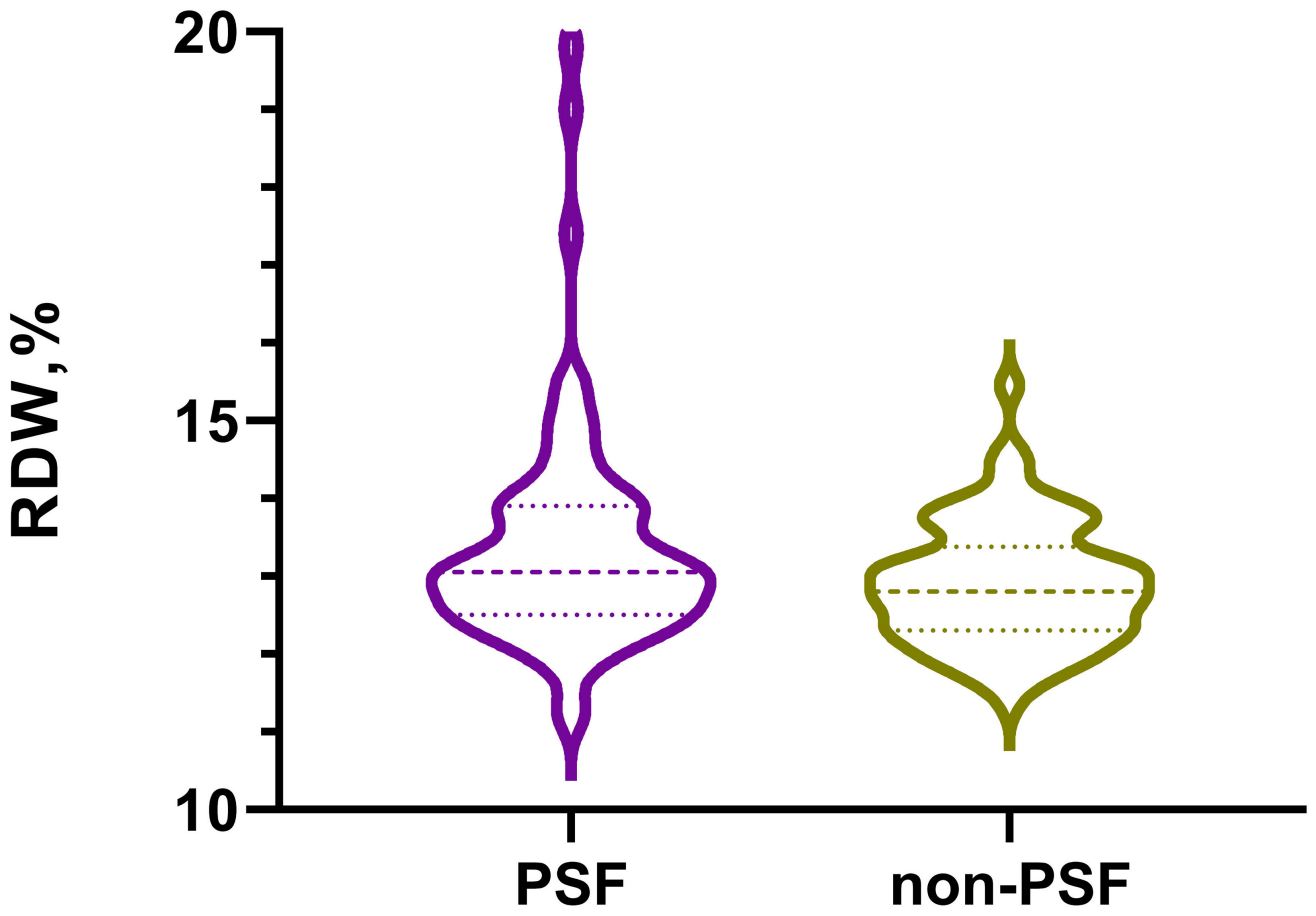
The violin plot in distribution of RDW between the PSF group and non-PSF group. RDW, red blood cell distribution width; PSF, post-stroke fatigue.

Table 1 showed the baseline data of AIS patients stratified by PSF or non-PSF. In our study, the patients in the PSF group (*n* = 70) were more likely to have higher proportion of coronary heart disease (*P* = 0.026), elevated levels of NIHSS score (*P* = 0.036), HAMA score (*P* = 0.001) and RDW (*P* = 0.023). Figure 2 displayed the levels of RDW between the PSF groups and non-PSF groups (13.1% [12.5, 13.9%] vs. 12.8% [12.3, 13.4%], *P* = 0.023).

**Table 1:** Baseline data of AIS patients stratified by PSF or non-PSF.

**Variable**	**PSF group (*****n** =* **70)**	**Non-PSF group** **(*****n** =* **136)**	***P***
**Demographics**			
Age, year	70.8 ± 10.6	68.5 ± 10.7	0.147
Male, *n* (%)	35 (50.0)	74 (54.4)	0.548
**Past medical history, *n* (%)**			
Hypertension	50 (71.4)	93 (68.4)	0.653
Diabetes	26 (37.1)	46 (33.8)	0.636
Cancer	3 (4.3)	9 (6.6)	0.755
Coronary heart disease	13 (18.6)	11 (8.1)	0.026
Atrial fibrillation	6 (8.6)	10 (7.4)	0.757
Arthritis	2 (2.9)	8 (5.9)	0.500
Previous stroke	21 (30.0)	28 (20.6)	0.133
**Tobacco use**			0.478
Never	15 (21.4)	28 (20.6)	
Ever	17 (24.3)	24 (17.6)	
Always	38 (54.3)	84 (61.8)	
**Alcohol Abuse**			0.356
Never	17 (24.3)	46 (33.8)	
Ever	9 (12.9)	17 (12.5)	
Always	44 (62.9)	73 (53.7)	
**Clinical data**			
SBP, mmHg	144.2 ± 19.9	142.5 ± 17.0	0.518
DBP, mmHg	82.7 ± 12.4	83.2 ± 14.1	0.814
NIHSS, score	2 (1, 3)	1 (1, 2)	0.036
HAMA, score	7 (4, 10)	3 (0, 6)	0.001
**Laboratory parameters**			
FBG, mmol/l	5.39 (4.79, 7.01)	5.33 (4.83, 6.99)	0.828
TC, mmol/l	4.14 (3.31, 5.00)	4.32 (3.69, 4.94)	0.371
TG, mmol/l	1.39 (0.99, 1.76)	1.49 (1.08, 2.00)	0.193
HDL, mmol/l	1.06 (0.92, 1.19)	1.03 (0.91, 1.19)	0.744
LDL, mmol/l	2.44 (1.87, 3.22)	2.64 (2.23, 3.25)	0.229
Creatinine, μmoI/L	73.0 (60.5, 86.5)	69.0 (59.6, 86.4)	0.777
RDW, %	13.1 (12.5, 13.9)	12.8 (12.3, 13.4)	0.023

AIS, Acute ischemic stroke; PSF, Post-stroke fatigue; SBP, Systolic blood pressure; DBP, Diastolic blood pressure; NIHSS, National institute of health stroke scale; HAMA, Hamilton anxiety scale; FBG, Fast blood glucose; TC, Total cholesterol; TG, Triglycerides; HDL, High density lipoprotein; LDL, Low density lipoprotein; RDW, Red blood cell distribution width.

**Figure 3:**
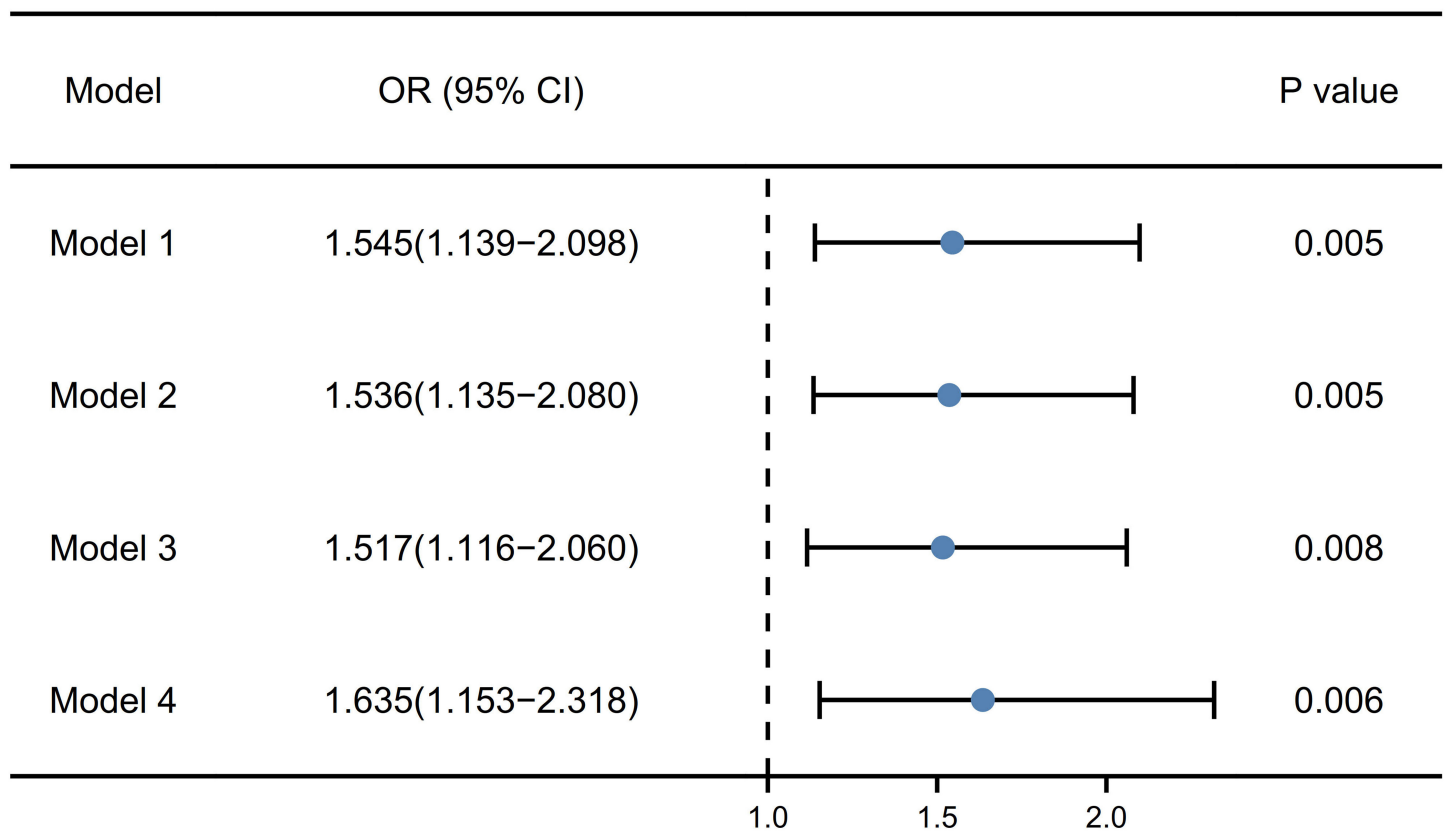
Multivariate logistic regression models for the association of RDW with PSF. Model 1, unadjusted model; Model 2, adjusted for age and sex; Model 3, adjusted for age, sex, coronary heart disease and baseline NIHSS score; Model 4, adjusted for age, sex, coronary heart disease, NIHSS score and HAMA score. RDW, red blood cell distribution width; PSF, post-stroke fatigue; NIHSS, National institute of health stroke scale; HAMA, Hamilton anxiety scale.

Figure 3 exhibited the results of logistic regression to explore the relationship between RDW and PSF in the acute phase. In the unadjusted model (model 1), RDW at admission might be related to PSF (OR [odds ratio], 1.545; 95% CI [confidence interval], 1.139–2.098; *P* = 0.005). After the adjustment for age and sex (model 2), RDW might also be the related factor to PSF (OR, 1.536; 95% CI, 1.135–2.080; *P* = 0.005). After the adjustment for age, sex, coronary heart disease and baseline NIHSS score (model 3), the OR of PSF for RDW was 1.517 (95% CI, 1.116–2.060, *P* = 0.008). What is more, in the model 4, which included age, sex, coronary heart disease, NIHSS score and HAMA score, RDW remained the independent associated factor with PSF (OR, 1.635; 95% CI, 1.153–2.318; *P* = 0.006).

**Figure 4:**
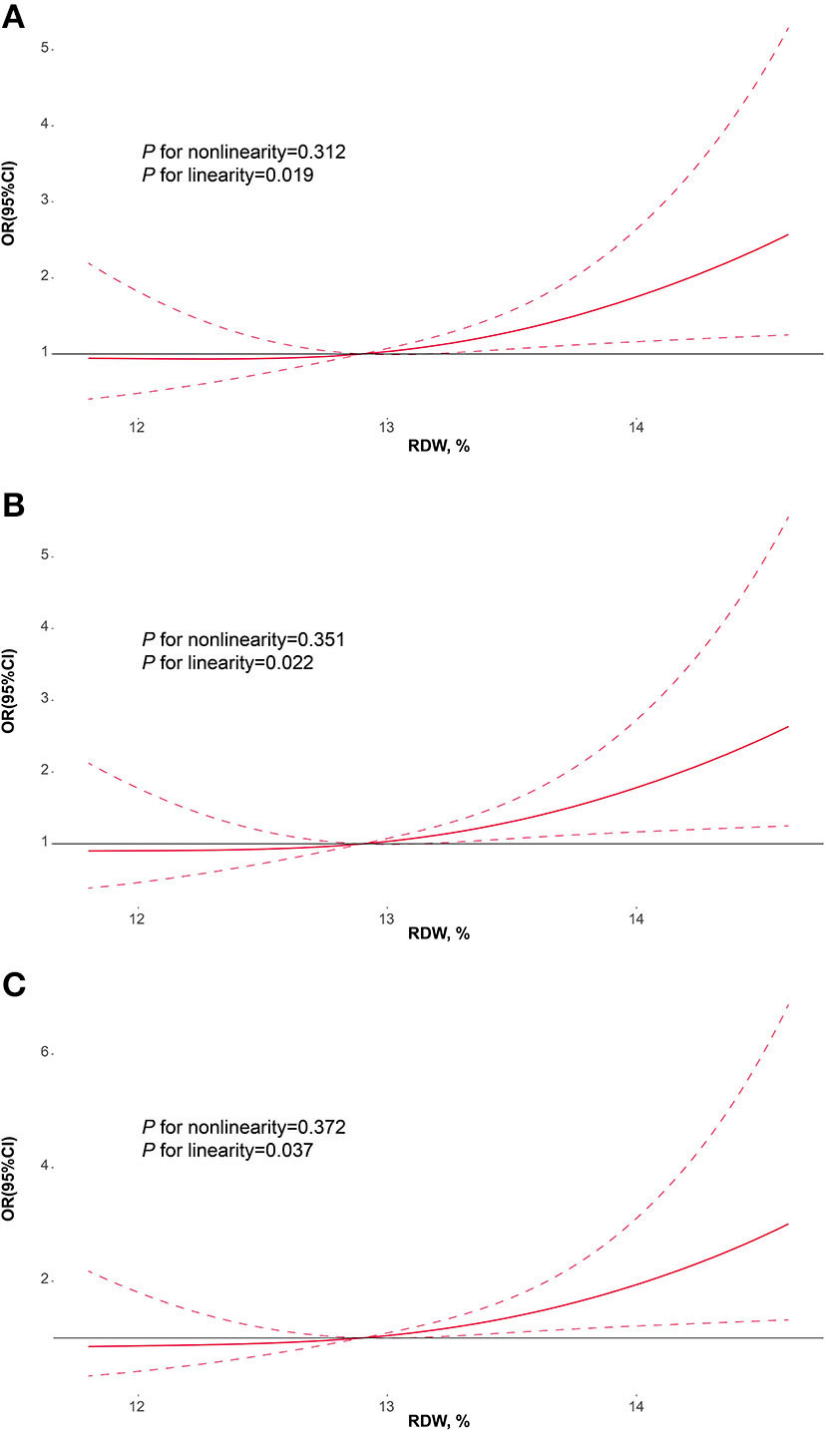
Restricted cubic spline regression models for the association of RDW with PSF. Adjusted odds ratios and 95% confidence intervals derived from restricted cubic spline regression, with knots placed at the 10th, 50th, and 90^th^ percentiles of RDW. **(A)** adjusted for age and sex; **(B)** adjusted for age, sex, coronary heart disease and baseline NIHSS score; **(C)** adjusted for age, sex, coronary heart disease, NIHSS score and HAMA score. RDW, red blood cell distribution width; PSF, post-stroke fatigue; NIHSS, National institute of health stroke scale; HAMA, Hamilton anxiety scale.

Figure 4 manifested the results of multivariable-adjusted spline regression models. In the model including age and sex, the linear dose-response associations of RDW at admission with PSF in the acute phase were found (*P* for non-linearity = 0.312; *P* for linearity = 0.019; Figure 4A). Furthermore, the linear dose-response associations of RDW with PSF in the acute phase remained significant in the model including age, sex, coronary heart disease and baseline NIHSS score (*P* for non-linearity = 0.351; *P* for linearity = 0.022; Figure 4B) and the model including age, sex, coronary heart disease, NIHSS score and HAMA score (*P* for non-linearity = 0.372; *P* for linearity = 0.037; Figure 4C).

## Discussion

In this prospective observational study, we have found that RDW at admission could be one independent associated factor with PSF in the acute phase of AIS, according to the results of different logistic regression models. In addition, based on the RCS models, the linear dose-response associations of RDW with PSF in the acute phase were confirmed.

The incidence of PSF in the acute phase is 34.0 % in our study, which is in line with previous studies (7–11). This may be attributed to our rigorous and meticulous assessment about PSF during this prospective study. The incidence also indicated that approximately 1/3 of AIS patients might suffer from PSF in the acute phase. Therefore, the neurologists may be supposed to pay attention to the management of PSF in the acute phase.

RDW is a readily available laboratory parameter, which could reflect the variability in volumes of red blood cell. Higher levels of RDW mean greater variation in volumes. In normal Chinese population, the levels of RDW range from 11.0 to 16.0%, and may increase under pathological conditions. It is well known that inflammatory response plays a prominent role on the pathophysiology of cerebrovascular disease (17–22). RDW may be associated with C-reactive protein, interleukin −6 and other inflammatory biomarkers (23, 24). The research performed by Semba RD et al. manifested that serum selenium might be an independent predictor of RDW and mediate effects on RDW *via* interleukin-6 (25). These findings showed that RDW might be able to be an inflammatory biomarker and be involved in the pathophysiology of several inflammation-related diseases, for example, cerebrovascular diseases.

Previous clinical studies have revealed the role of RDW on cerebrovascular disease. Vayá A et al. found that RDW >14.0% might increase the risk of cryptogenic stroke (26). The results of one cross-sectional study, which enrolled 432 primary AIS patients, indicated that the AIS patients with carotid artery atherosclerosis could possess higher levels of RDW (27). RDW may also serve as an independent related factors to the prognostic outcomes in AIS patients treated with intravenous thrombolysis (28). Another research recruited the AIS patients without intravenous thrombolysis or endovascular treatment, and showed that elevated levels of RDW were related to increased risk of hemorrhagic transformation (29). A meta-analysis displayed that the baseline levels of RDW might be a predictor of stroke occurrence and outcome (30). What is more, high RDW levels may increase the risk of hemorrhagic transformation after intravenous thrombolysis in AIS patients (31). Nowadays, the levels of RDW were found to be linked to post-stroke depression (16), another common emotional complication after the onset of AIS. Although there are several studies focusing on the relationship between RDW and cerebrovascular disease, this study is the first prospective study that explore the role of RDW on PSF, and may assist neurologists with managing AIS patients.

However, there are still several shortcomings in our prospective observational study. First, we only collected the data about PSF in the acute phase now. Therefore, we are following up the PSF at 6 months in these AIS patients prospectively, and we will explore the relationship between RDW and PSF at 6 months in the future research. Second, the sample size of this study is relatively small. We aim to carry out the study with large sample to provide higher levels of evidence about the association of RDW with PSF. Third, RDW and other laboratory parameters may change during hospitalization, so it might be critical to monitor these parameters, dynamically. In addition, we have not utilized machine learning in this study and collected the data of other inflammatory biomarkers, such as interleukin-6 (IL-6) and tumor necrosis factor-α (TNF-α).

In short, this prospective observational study is the first study centering on the relationship between RDW at admission and PSF in the acute phase of AIS, as far as we know. Linear dose-response associations of RDW with PSF in the acute phase of AIS have been found. Consequently, RDW at admission may be a novel biomarker of PSF in the acute phase, which is helpful for neurologists. Nevertheless, the study with large sample is required in the future, and these conclusions need to be verified in other stroke centers.

## Data availability statement

The original contributions presented in the study are included in the article, further inquiries can be directed to the corresponding author.

## Ethics statement

The studies involving human participants were reviewed and approved by the Ethics Committee of Nantong Third People's hospital (Ethics number: EK2021008). The patients/participants provided their written informed consent to participate in this study.

## Author contributions

MP wrote the manuscript and performed the statistical analyses. YuC collected the data and assisted with writing the manuscript. YaC, KF, HS, HH, WZ, HZ, and JJ collected the data. All authors contributed to the article and approved the submitted version.

## Funding

This study was supported by Social and People's Livelihood Science and Technology Innovation Special Project in Nantong City (ms12021065).

## Conflict of interest

The authors declare that the research was conducted in the absence of any commercial or financial relationships that could be construed as a potential conflict of interest.

## Publisher's note

All claims expressed in this article are solely those of the authors and do not necessarily represent those of their affiliated organizations, or those of the publisher, the editors and the reviewers. Any product that may be evaluated in this article, or claim that may be made by its manufacturer, is not guaranteed or endorsed by the publisher.
